# Prediction of pathologic complete response to neoadjuvant chemoradiation in locally advanced rectal cancer

**DOI:** 10.3389/fonc.2024.1361300

**Published:** 2024-03-11

**Authors:** Xiaoling Zhong, Guohua Zeng, Lixiang Zhang, Shuyuan You, Yuxiang Fu, Wan He, Guixiang Liao

**Affiliations:** ^1^ Department of Radiology, Shenzhen People's Hospital (The Second Clinical Medical College of Jinan University, the First Affiliated Hospital of Southern University of Science and Technology), Shenzhen, Guangdong, China; ^2^ Department of Pathology, Shenzhen People's Hospital (The Second Clinical Medical College of Jinan University, the First Affiliated Hospital of Southern University of Science and Technology), Shenzhen, China; ^3^ Department of Gastrointestinal surgery, Shenzhen People's Hospital (The Second Clinical Medical College of Jinan University, the First Affiliated Hospital of Southern University of Science and Technology), Shenzhen, China; ^4^ Department of Oncology, Shenzhen People's Hospital (The Second Clinical Medical College of Jinan University, the First Affiliated Hospital of Southern University of Science and Technology), Shenzhen, China; ^5^ Department of Radiation Oncology, Shenzhen People's Hospital (The Second Clinical Medical College of Jinan University, the First Affiliated Hospital of Southern University of Science and Technology), Shenzhen, China

**Keywords:** LARC, NCRT, PCR, MRI, tumor volume, tumor differentiation

## Abstract

**Purpose:**

To investigate the predictive factors of pathologic complete response (pCR) in locally advanced rectal cancer (LARC) patients who had been treated with neoadjuvant chemoradiation (nCRT).

**Methods and materials:**

For this retrospective study, 53 LARC patients (37 males and 16 females; age range 25 to 79 years) were selected. Clinical characteristics, baseline mrTNM staging, MR gross tumor volumes (GTV), and pCR were evaluated. The diagnostic accuracy of GTV for predicting pCR was calculated.

**Results:**

Among 53 LARC patients, 15 patients achieved pCR (28.3%), while 38 patients achieved non-pCR. Only three (5.7%) out of 53 patients did not downstage after nCRT. GTV and tumor differentiation were the significant prognostic parameters for predicting pCR. A tumor volume threshold of 21.1 cm^3^ was determined as a predictor for pCR, with a sensitivity of 84% and specificity of 47%. In addition, GTV was associated with mrN stage, circumferential resection margin (CRM) status, extramural vascular invasion (EMVI) status, and pretreatment serum CEA level.

**Conclusion:**

Tumor volume and tumor differentiation have significant predictive values in preoperative assessment of pCR among LARC patients. These findings aid clinicians to discriminate those patients who may likely benefit from preoperative regimens and to make optimal treatment plans.

## Introduction

Rectal cancer is a common cancer worldwide (1). Due to the widespread use of rectal magnetic resonance imaging (MRI), radiologists’ understanding of the main MRI features of rectal cancer, early detection, and improved treatment of rectal cancer, the prognosis of rectal cancer has improved in recent decades (2). However, about half of patients are diagnosed with locally advanced cancer (LARC), which has a higher rate of recurrence and mortality (3). The application of neoadjuvant radiochemotherapy (nCRT) and total rectal mesorectal excision (TME) as a standard treatment has improved the local control for rectal cancer (4). Almost half of patients with LARC After nCRT neoplasms may decrease in stage and one-third of tumors showing pathological complete response (pCR) while TMD TME surgery performed (5, 6). Compared to patients without PCR, those with pCR are related with a better prognosis in local control, distant recurrence, disease-free survival (DFS), and overall survival (OS) (6, 7). A study indicated that an observation approach for LARC after a clinical complete response (cCR) showed no significant differences in non-regrowth cancer recurrence or OS rate between observational and surgical patients (8), which means most cCR patients can avoid the morbidity of radical surgery. Lord et al. evaluated NICE criteria for preoperative radiotherapy in patients with rectal cancer treated only surgically in 2020 and compared them with confirmed MRI prognostic factors. They found that confirmed MRI prognostic factors (extramural venous invasion, tumor deposition, and peripheral margin) were better able to identify high-risk groups (9).

Up to now, accurately predicting pCR or non-pCR to nCRT still remains a challenge, even though it is a crucial prerequisite for making appropriate treatment decisions about whether to make a watch-and-wait strategy for cCR patients, or to intensify treatment for those non-cCR. Therefore, this study attempted to investigate potential preoperative clinical and MRI markers to identify tumor response to nCRT and non-response among LARC patients, thus assisting in determining the optimal treatment planning.

## Materials and methods

### Patients

**Figure 1:**
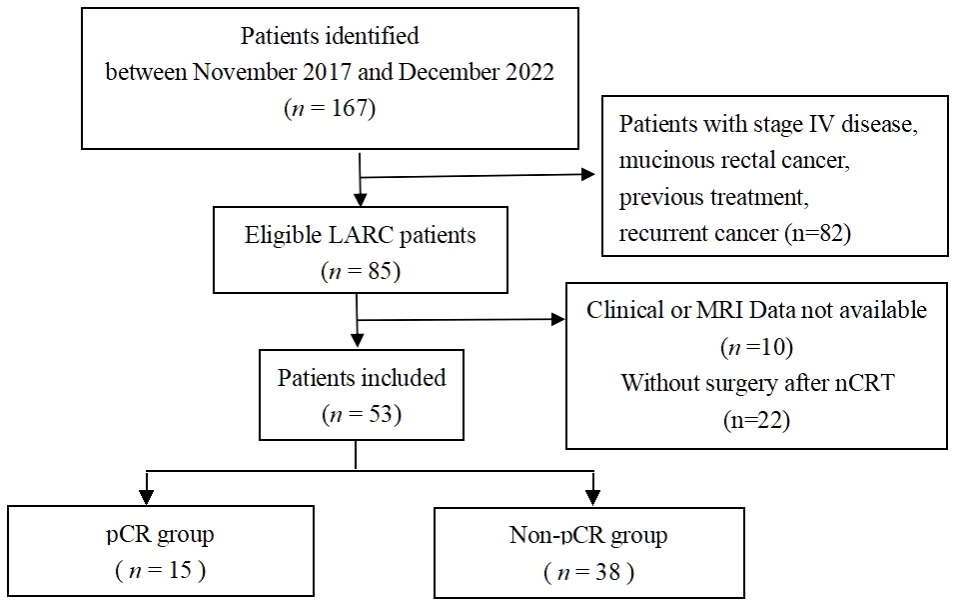
Study flow diagram.

We retrospectively analyzed the data of consecutive rectal cancer patients between November 2017 and December 2022. Subsequently, 53 rectal cancer patients who were confirmed by surgical pathology and met the following criteria were enrolled: (a) histopathologically confirmed as rectal adenocarcinoma; (b) diagnosed as LARC, which was defined as clinical stage II (T3/4, node negative) or stage III (node positive) before treatment; (c) had evaluable MR imaging before nCRT; (d) had complete nCRT that was followed by surgical treatment after 5-12 weeks; (e) had complete clinical history; and (f) was free of induction or consolidation chemotherapy before or after the chemoradiation course. The exclusion criteria were patients with stage IV disease, mucinous rectal cancer, previous treatment, recurrent cancer, unavailable clinical or MRI data, or without surgery after nCRT. The process of patients’ selection is listed in **Figure 1**.

### MR examination and image analysis

1.5T or 3.0T MR were performed for each patient. Scanners used a pelvic phased-array coil. Patients were not asked to undergo bowel preparation and did not receive anti-peristaltic medication before MRI. Standard T2-weighted image (T2WI) fast spin-echo sequences, including sagittal and axial (perpendicular to the long axis of the intestinal lesion), were performed. Diffusion-weighted imaging (DWI) and contrast enhanced T1-weighted image (CE-T1WI) axial scans were also carried out. The acquisition parameters of MRI scans derived from different devices are summarized in the data supplement. After the collection of MR image data, they were sent to the PACS system.

Rectal cancer MRI staging was based on the American Joint Committee on Cancer (AJCC) TNM staging system for colorectal cancer (8th edition in 2016) (10) and Horvat N et al. (11). The tumor’s pretreatment baseline mrTNM staging was evaluated by two board-certified radiologists independently, including tumor location (distance from the anal verge), T category, N category, circumferential resection margin (CRM) status, extramural vascular invasion (EMVI) status, and tumor deposit (TD) status. If there was any disagreement, consensus was reached after the discussion.

**Figure 2:**
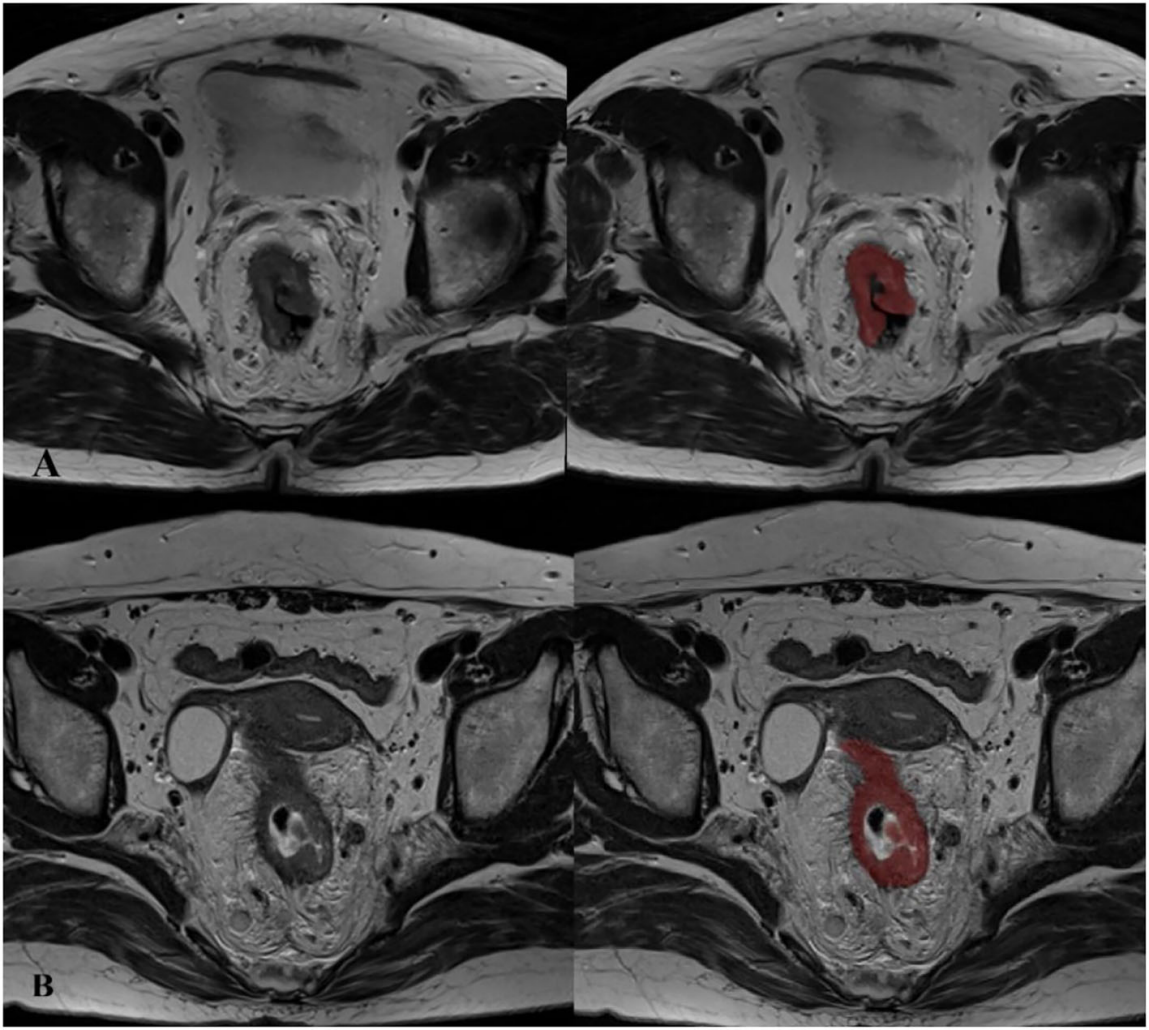
Examples for gross tumor volumetry in rectal cancer on axial contiguous MR images. **(A)** A 69-year-old man with a tumor in the mid-rectum, before treatment staged as T3N2, EMVI (+), CRM (+), TD (-). **(B)** A 57-year-old woman with a tumor in the low-rectum, before treatment staged as T4N2, EMVI (+), CRM (+), TD (+).

The pretreatment gross tumor volume (GTV) was carried out using the ITK-SNAP tool (Version 3.8.0. The tumor on the MR-T2WI axial oblique images was contoured manually slice by slice; non-involved soft tissues, feces, and central lumen were avoided. The GTV measurements were conducted by a single board-certified radiologist and finally revised by another experienced radiologist. The unit of GTV is cm^3^. **Figure 2** shows representative MRIs.

### Neoadjuvant chemoradiotherapy

All patients received concurrent chemoradiotherapy based on oral capecitabine, starting on the first day of radiotherapy, with a dose of 1650 mg/m^2^/d, divided into morning and evening doses, continuing until the end of radiotherapy. And mFOLFOX6 (oxaliplatin, 5-fluorouracil and calcium folinate) was used as adjuvant chemotherapy. Rectal irradiation was given by lateral opposed fields to the whole pelvis delivered by Varian (6MV). The radiation dose was 50.4Gy in 27 fractions, with 5 days’ treatment per week.

### Surgery and pathological TRG category

All patients underwent TME surgery 5-12 weeks after nCRT, based on further examinations confirming no surgical contraindications. All postoperative pathology specimens were determined by our hospital’s pathology department.

**Figure 3:**
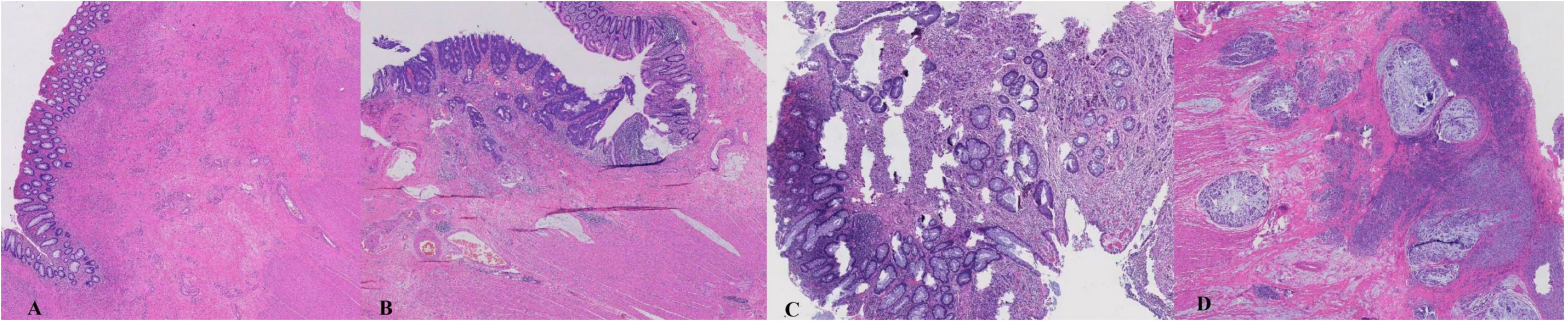
Tumor regression in rectal surgical specimens after neoadjuvant chemoradiation. **(A)**: TRG 0, pathological complete response; **(B)**: TRG 1; **(C)**: TRG 2; **(D)** TRG 3.

The pathological tumor regression grade (TRG) was based on the classification standard of the American Joint Committee on Cancer/College of American Pathologists (AJCC/CAP) system (12). It is divided into four categories: TRG 0, no tumor cells visible under the microscope; TRG 1, only a single or small cluster of tumor cells remaining; TRG 2, tumor residual with predominant fibrosis; and TRG 3, none or small amount of tumor cell necrosis, extensive tumor residue. Define TRG 0 as the pCR group and TRG 1-3 as the non-pCR group. The TRG category data were independently defined by two experienced pathologists. **Figure 3** demonstrates tumor regression in rectal surgical specimens after nCRT.

### Statistical analysis

SPSS software (Version 25.0 IBM Corp, Armonk, NY, USA) was adopted. Descriptive statistics such as mean with standard deviation were used for continuous data. Independent *t* tests, Wilcoxon rank-sum tests, or Mann-Whitney tests as appropriate were used to compare continuous variables, while the χ^2^ tests were used to compare categorical data. Receiver operating characteristic (ROC) analysis was performed to calculate diagnostic sensitivity and specificity of GTV that predicts for pCR, and a cutoff value was established according to Youden’s *J* test. *P* values of.05 were considered statistically significant.

## Results

### Characteristics of patents

Among fifty-three LARC patients who met the inclusion criteria in this study, there were 37 men (69.8%) and 16 women (30.2%). The mean age was 56.9 ± 12.2 years (range, 25-79). The pretreatment median tumor volume was 33.7cm^3^ (range, 5-233cm^3^), and the median distance from the anal verge evaluated by MRI was 4.5cm (range, 1.3-9cm). Pretreatment clinical assessment demonstrated only one stage II (1.9%) and 52 stage III (98.1%). Confirmed by surgical pathology after treatment, 15 (28.3%) patients had TRG 0, while 14 (26.4%), 20 (37.8%), and four (7.5%) patients had TRG 1, 2, and 3 respectively. That is, 15 (28.3%) out of 53 patients included in this study achieved pCR, while 38 (71.7%) patients achieved non-pCR. The clinical and pretreatment characteristics of LARC patients in this study are shown in **Table 1**.

**Table 1:** Characteristics of 53 patients with locally advanced rectal cancer.

	pCR (n=15), n (%)	Non-pCR (n=38), n (%)	*P* value
Sex			0.333
Female	6 (40)	10 (26.3)	
Male	9 (60)	28 (73.7)	
Age (y)			0.114
Mean ± SD	52.7 ± 13.6	58.6 ± 11.4	
CEA level			0.230
Normal	11 (73.3)	21 (55.3)	
Abnormal	4 (26.7)	17 (44.7)	
Gross tumor volume (cm^3^)			0.040^⁎^
Mean ± SD	27.0 ± 14.0	46.8 ± 40.7	
Tumor location (from anal verge)			0.765
Low	8 (53.3)	22 (57.9)	
Middle	7 (46.7)	16 (42.1)	
High	0	0	
Tumor differentiation			0.000^⁎^
Poor	1 (6.7)	11 (28.9)	
Moderate	5 (33.3)	24 (63.2)	
Well	9 (60)	3 (7.9)	
cTNM stage at baseline			0.111
I-II	1 (6.7)	0	
III	14 (93.3)	38 (100)	
mrT stage at baseline			0.072
T1-2	2 (13.3)	2 (5.3)	
T3	11 (73.4)	22 (57.9)	
T4	2 (13.3)	14 (36.8)	
mrN stage at baseline			0.483
N0-1	2 (13.3)	9 (23.7)	
N2	13 (86.7)	29 (76.3)	
mrTD status at baseline			0.243
Negative	11 (73.4)	33 (86.8)	
Positive	4 (26.7)	5 (13.2)	
mrEMVI status at baseline			0.314
Negative	2 (13.3)	10 (26.3)	
Positive	13 (86.7)	28 (73.7)	
mrCRM status at baseline			0.509
Negative	4 (26.7)	7 (18.4)	
Positive	11 (73.3)	31 (81.6)	

pCR, pathologic complete response; SD, standard deviation; TD, tumor deposit; EVMI, extramural vascular invasion; CRM, circumferential resection margin; CEA, carcinoembryonic antigen.

*Signifies a significant difference between pCR and non-pCR groups (P < 0.05).

### Pretreatment clinical factors between the pCR and non-pCR patients

The mean age was 52.7 ± 13.6 years for pCR group and 58.6 ± 11.4 years for non-pCR group. No significant differences were found between these two groups in terms of age, gender, pretreatment clinical TNM staging, and CEA level, while tumor differentiation was the significant difference between these two groups (*P* = 0.000) (**Table 1**), namely that well differentiated rectal carcinomas seemed to attain a better tumor response and higher pCR rate.

### Pretreatment MRI status between the pCR and non-pCR patients

**Figure 4:**
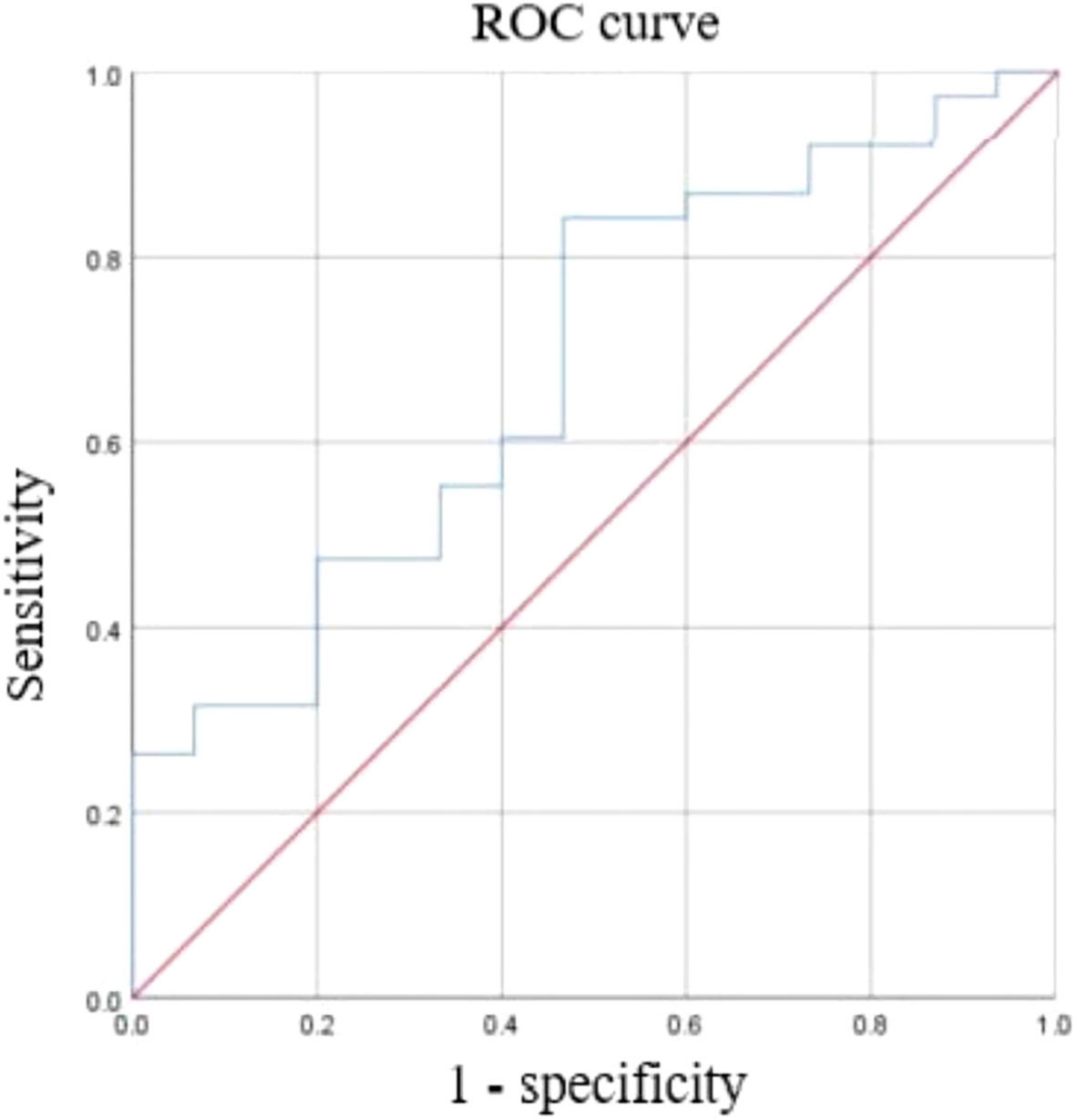
Receiver operating characteristic (ROC) curve with tumor volume cutoff threshold for pathological complete response. Area under the curve (AUC) = 0.682, 95% CI: 0.525, 0.84.

Pretreatment mrT stage, mrN stage, tumor location, GTV, CRM, EMVI, and TD status were analyzed for prediction of pCR; GTV was the only statistically significant factor (*P* = 0.04) (**Table 1**). Subsequently, ROC analysis of GTV showed that the area under the curve value was 0.68 with asymptotic significance level (*P* = 0.04) and the tumor volume threshold was 21.1 cm^3^ (**Figure 4**), which showed a sensitivity of 84% and specificity of 47% for predicting pCR (95% CI: 0.525, 0.840).

In addition, GTV was associated with mrN stage, CRM, EMVI, and pretreatment serum CEA level among all 53 LARC patients (**Table 2**). However, in the non-pCR group, GTV was just associated with CRM and EMVI; no statistically significant association between GTV and other parameters except tumor location was observed in the pCR group.

**Table 2:** Factors associated with gross tumor volume.

	53 LARC patients and *P* value	pCR group and *P* value	Non-pCR group and *P* value
mrT stage at baseline	0.15	0.47	0.47
mrN stage at baseline	0.03	0.09	0.06
mrTD status at baseline	0.85	0.36	0.65
mrEMVI status at baseline	0.006	0.23	0.005
mrCRM status at baseline	0.002	0.24	0.007
CEA level	0.04	1	0.06
Tumor location (from anal verge)	0.35	0.04	0.84
Tumor differentiation	0.22	0.4	0.74

### Posttreatment ypTN status between the pCR and non-pCR patients

After treatment, all 15 pCR patients reached ypT0N0. In the non-pCR group, there were 5/38 (13.1%) patients with ypT1 stage, 13/38 (34.2%) patients with ypT2 stage, 18/38 (47.4%) patients with ypT3 stage, and 2/38 (5.3%) patients with ypT4 stage; there were 26/38 (68.4%) patients with ypN0 stage, 10/38 (26.3%) patients with ypN1 stage, and 2/38 (5.3%) patients with ypN2 stage. In the non-pCR group, there were 12/38 (31.6%) stage I patients, 14/38 (36.8%) II patients, and 12/38 (31.6%) III patients. Only 3/53 (5.7%) patients did not downstage. The posttreatment characteristics of LARC patients in this study are shown in **Table 3**.

**Table 3:** Characteristics of patients in the pCR and non-pCR groups after treatment.

	pCR (n=15), n (%)	Non-pCR (n=38), n (%)
ypTNM stage		
I	NA	12 (31.6%)
II	NA	14 (36.8%)
III	NA	12 (31.6%)
ypT status		
T0	NA	0
T1	NA	5 (13.1%)
T2	NA	13 (34.2%)
T3	NA	18 (47.4%)
T4	NA	2 (5.3%)
ypN status		
N0	NA	26 (68.4%)
N1	NA	10 (26.3%)
N2	NA	2 (5.3%)

## Discussion

In the current study, we found some evidence that gross tumor volume (GTV) and tumor differentiation were the significant prognostic parameters for predicting pCR. And we have found that pCR was in a rate of 28.3% among LARC patients treated with nCRT, which was similar to previous research. Factors such as age, gender, tumor location from anal verge, pretreatment CEA level, clinical TNM stage, and MRI parameters including mrTD, mrEMVI, and mrCRM failed to predict pCR. Additionally, GTV was associated with mrN stage, mrCRM, mrEMVI, and pretreatment serum CEA level among all the patients. These results help select individuals who may likely benefit from preoperative therapy.

The prediction of pCR in LARC patients has always been challenging. In earlier research, De Felice et al. found that pretherapeutic tumor size less than 5 cm could be considered as a significant predictor for pCR (13). And Reggiani et al. revealed tumor length larger than 3cm would be an independent prognostic factor, which tended to have worse DFS and cancer-specific survival (CSS) (14). Jankowski et al. argued that watch-and-wait strategy in patients with tumor length more than 7 cm was undetermined (15). However, it is easy to measure tumor length while not comprehensively reflecting the characteristics of tumor itself.

Recently, several studies have elucidated the value of tumor volume in predicting prognosis. Martens et al. reviewed literature on tumor measurements on MRI and validated tumor length or 3-dimensional tumor size were not accurate enough to assess the tumor response after chemoradiotherapy (16). They found tumor volume measured by MR achieved up to 80% accuracy to assess a complete tumor response. Jiang et al. reported a tumor volume less than 9.49 cm^3^ was significantly correlated with DFS and local recurrence-free survival (LRFS) in earlier rectal cancer patients who had been operated on with radical surgery (17). And the tumor volume was significantly associated with pretherapeutic CEA level, Hb level, and the number of lymph nodes. Lutsyk et al. found tumor volume less than 39.5 cm^3^ was a significant predictor for achieving pCR among 187 LARC patients (18). Similarly, Yang et al. demonstrated tumor volume less than 37.3 cm^3^ could be predictive for pCR in 412 LARC patients receiving nCRT (19).

Considering the tumor volumetry might result in bias from different MR devices, we used a new computational algorithm, which is based on MRI spatial voxels, that makes a more precise measurement of GTV. This voxel-based approach for GTV utilizing MR scans is an improvement from conventional rough 1-dimensional and 3-dimensional tumor measurements, regardless of volume data from different modalities. To some extent, this may be the reason why tumor volume in our study was smaller than other studies. Moreover, Maas et al. had compared the accuracy of 3T and 1.5T MR scanners to discriminate between T2 and borderline T3 rectal cancers when performing exams on the same group of patients and found no significant differences between the two MRI scanners (20), which suggested that it could not be a confounder impacting the tumor volume estimation. And all the patients in our study were performed on a standardized imaging protocol, which allows for accurate and reproducible interpretations in the evaluation of rectal cancer.

Tumor differentiations are found more frequently to associate with prognosis. Poor differentiated tumors are more commonly found to be aggressive, by invading blood vessels and nerves and adjacent histological boundaries. Al-Sukhni et al. identified lower tumor grade was correlated with higher odds of pCR among 23,747 patients with rectal cancer who received nCRT (21). A recent retrospective study of 325 patients demonstrated that poor differentiation was recognized as an independent risk factor for tumor local recurrence and 3-year overall survival (22). However, Huang et al. did not discuss the assessments of the tumor itself, neither the length nor volume. Reggiani et al. also suggested that a comprehensive approach should be applied to rectal cancer patients with poor differentiation (14).

Some studies have identified the significance of several clinical and radiological markers in predicting pCR and non-pCR among LARC patients. Huh JW et al. reported that pretreatment tumor circumference, tumor ulceration, and CEA level should be considered when attaining pCR (23). However, they did not evaluate pretreatment tumor volume. Zhao et al. used mrDEC score to predict tumor response to nCRT and showed that mrTDs and mrEMVI were statistically significant but not mrCRM and mrDEC (24). We also tried to use mrDEC scoring system to detect pCR but found no significant difference in our study. In addition to the commonly used comprehensive assessments, functional imaging is also increasingly being applied to evaluate prediction in LARC patients. Lambregts et al. demonstrated that diffusion-weighted MRI (DWI) helped to identify complete tumor response after CRT by qualitative evaluation (25). Joe et al. systematically reviewed the data on the role of DWI and ^18^F-FDG PET/CT when attaining pCR, and they revealed that DWI and ^18^F-FDG PET/CT were not accurate enough to stratify patients for conservative approaches (26). Lian et al. found that the mean T1 and T2 values were significantly lower in pCR patients and those T-downstage patients by pretreatment quantitative synthetic MRI (27). However, proton density (PD) and ADC values failed to identify pCR and T-downstaging. Iafrate et al. showed that pretreatment ADC values were significantly lower in pCR patients when compared with those non-pCR patients, but they failed to identify pretreatment tumor volume associated with pathological response, with the median value of 21.3 cm^3^ and 24 cm^3^ respectively (28). In the current study, we did not evaluate ADC values as it was generated by variate equipment which might be unreliable.

Research based on radiomics has been emerging recently to evaluate the tumor response to nCRT in LARC patients. Zhou et al. indicated that pretreatment, multiparametric MRI radiomic features played an important role in predicting non-response to nCRT (29). Ren et al. developed nomograms for predicting pCR probability and showed the significance of neoadjuvant therapy options, tumor differentiation, MRF status, and tumor length (30). Chiloiro G et al. and Shin J et al. used radiomics models and showed a good performance for predicting pCR after nCRT (31, 32). Moreover, Chiloiro G et al. found that the best performing two-year DFS prediction model was developed on the basis of tumor volume as well as mesorectal features (33).

This study was limited by its small sample size and retrospective nature. When assessing pCR in LARC patients, lymph node status did not play a significant predictive role in this study, which is due to the fact that those enrolled LARC patients were mostly associated with lymph node positive when they arrived at our hospital, thus resulting in a similar preoperative lymph node status between the two groups. Furthermore, an increase in the number of T4 patients in the non-pCR group may interfere with the significance, which marginally showed no significant difference in T stage between the two groups. And we could not provide enough data to use multivariable logistic regression models to investigate factors that may have an independent influence on tumor response. If the dataset is small, it is not conducive to obtaining a better training mode when splitting the same dataset in training and evaluation subsets. This is why we did not run an external validation study. Regardless, these results in the current study have demonstrated potential predictors based on clinical characteristics and MRI markers. Further large and prospective studies are on the way to validate these findings.

## Conclusion

The current study shows that preoperative gross tumor volume and tumor differentiation can be potential predictors for pCR in LARC. These findings help clinicians to stratify those patients who may benefit from a conservative rather than aggressive therapeutic approach after nCRT. When evaluating the clinical response, clinicians can make a more personalized regimen for rectal cancer patients based on personal characteristics and patient’s risk factors.

## Data availability statement

The raw data supporting the conclusions of this article will be made available by the authors, without undue reservation.

## Ethics statement

The studies involving humans were approved by the ethics committee review board of Shenzhen People’s Hospital. The studies were conducted in accordance with the local legislation and institutional requirements. Written informed consent for participation in this study was provided by the participants’ legal guardians/next of kin. Written informed consent was obtained from the individual(s) for the publication of any potentially identifiable images or data included in this article.

## Author contributions

XZ: Conceptualization, Data curation, Formal analysis, Methodology, Validation, Writing – original draft, Writing – review & editing. GZ: Data curation, Formal analysis, Writing – original draft. LZ: Data curation, Formal analysis, Writing – original draft. SY: Data curation, Formal analysis, Writing – original draft. YF: Data curation, Formal analysis, Investigation, Writing – review & editing. WH: Conceptualization, Investigation, Methodology, Writing – review & editing. GL: Conceptualization, Data curation, Formal analysis, Funding acquisition, Investigation, Supervision, Validation, Writing – original draft, Writing – review & editing.
